# Prioritising medication management criteria to identify emergency department patients with potential medication-related harm: Nominal Group Technique workshops with clinicians

**DOI:** 10.1007/s11096-026-02199-9

**Published:** 2026-07-31

**Authors:** Amber Franich, Kate Hill, Gary Grant, H. Laetitia Hattingh

**Affiliations:** 1 School of Pharmacy and Medical Sciences, Griffith University, Southport, QLD 4222 Australia; 2 Pharmacy, Gold Coast Hospital and Health Service, Southport, QLD 4215 Australia; 3 Allied Health Research, Gold Coast Hospital and Health Service, Southport, QLD 4215 Australia; 4 School of Pharmacy and Pharmaceutical Sciences, The University of Queensland, Brisbane, QLD 4102 Australia

**Keywords:** Consensus-based criteria, Emergency department, Medication-related harm, Medication review, Pharmacist

## Abstract

**Introduction:**

Pharmacist involvement in the emergency department (ED) and early in a patient’s hospital stay improves clinical outcomes and reduces medication errors. Because EDs are high-pressure settings with limited pharmacist staffing, pharmacists cannot review every patient. Instead, they must focus on identifying and prioritising patients who may be at risk of potential medication-related harm.

**Aim:**

To establish and prioritise consensus-based medication management criteria to identify patients with potential medication-related harm within the ED setting for early pharmacist review.

**Method:**

We used Nominal Group Technique (NGT) methodology to rank the most important criteria for identifying whether a patient’s presentation may be due to medication-related harm or whether they may be at risk of developing medication-related harm in the ED. Participants were purposively recruited ED clinicians including pharmacists, doctors and nurses who worked at the ED of either the Gold Coast University Hospital or Robina Hospital on the Gold Coast, Queensland, Australia, during May–June 2025.

**Results:**

A total of 14 participants comprising six pharmacists, five nurses and three doctors participated in two 1-h NGT workshops. Participants ranked 12 criteria from a list of 34 during NGT Round 1. They subsequently ranked six criteria from the list of 12 during NGT Round 2. The weighted rankings of the criteria across both groups were combined to determine the top six criteria which were, in order from 1 to 6 (1 = most relevant): (1) medication-related admission or thinks medicine responsible for admission; (2) bleeding and falls; (3) poor historian (unable to provide reliable history)/suspected non-adherence or poor compliance; (4) carbidopa/levodopa (entacapone), co-beneldopa; (5) unverified medicines; and (6) medicines requiring therapeutic drug monitoring/medicines with narrow therapeutic index. The first four criteria were ranked within the top six in both NGT workshops.

**Conclusion:**

This study has provided a concise list of six medication-related criteria that expert consensus agreed were the most important for prioritising patients with potential medication-related harm. This forms the basis for the development of information gathering strategies to be incorporated into clinicians’ workflows early in patients’ ED journey.

**Supplementary Information:**

The online version contains supplementary material available at https://doi.org/10.1007/s11096-026-02199-9.

##  Impact statements

- Through a structured consensus process, clinicians agreed on medication management criteria to identify emergency department patients at risk of potential medication-related harm.
- The criteria will enable the development of processes to facilitate targeting of patients with potential medication-related harm.
- Approaches to identify patients with potential medication-related harm will facilitate timely pharmacist review.

## Introduction

Medication-related harm is recognised as a major global issue that threatens patient safety. A 2023 systematic review of 100 studies involving 487,162 patients found that approximately 1 in 20 patients experience preventable medication-related harm, with one quarter of these events classified as severe or potentially life-threatening [1]. These findings underscore the urgent need for strategies that reduce preventable medication-related harm and improve the safety and quality of medicine use. Medication-related harm encompasses a wide range of problems related to medicines, including over- or under-use, inappropriate medicines, drug–drug interactions, and adverse effects [2]. It is strongly associated with polypharmacy, multiple comorbidities, and transitions of care [3]. Medication-related harm remains a frequent but preventable cause of emergency department (ED) presentations and hospital admissions [4].

Hospital pharmacists play a key role in identifying and reducing medication-related harm, particularly through activities such as medication history taking, medication reconciliation, and early detection of adverse medication-related events. Evidence consistently demonstrates that pharmacist involvement in the ED and early in the patient’s hospital journey improves clinical outcomes and reduces medication errors [5–9]. However, EDs are busy environments where pharmacist staffing is often limited hence pharmacists are not able to review all ED patients but rather need to prioritise those patients presenting with potential medication-related harm.

Triage tools are used by ED nurses to assess patients’ clinical urgency. For example, there are five triage categories within the Australian Triage Scale (ATS) with ATS1 most urgent (requiring immediate care) and ATS5 least urgent (requiring care within 60–120 min [10]. Once triaged by nurses, patients are reviewed by doctors and nurses and other health professionals, depending on their presentations. Tools for prioritising patients with possible medication-related harm in the ED have been developed and tested [11–18] and whilst some [11–13, 18] demonstrate high sensitivity, they have low specificity that could potentially result in large numbers of patients being identified who do not require early pharmacist intervention. Although these tools are useful, they include an extensive list of questions, between six and 15 questions, which may limit their practical application in a busy ED setting.

A 2023 study at Gold Coast Hospital and Health Service (GCHHS) explored processes/procedures followed by GCHHS ED clinicians to identify patients presenting due to medication-related harm [19]. Semi-structured interviews with five doctors, five pharmacists and five nurses found varied and inconsistent processes to identify patients who were either admitted with or suffered potential medication-related harm in the ED during admission. The clinicians identified a need for a structured process and localised tool within EDs to assist ED clinicians in identifying patients with potential medication-related harm for early pharmacist review. This subsequent study focused on establishing medication-related criteria to be incorporated into clinical decision processes or tools for future use in EDs to guide clinicians when they gather information from patients.

### Aim

The aim of this study was to establish and prioritise consensus-based medication management criteria to identify patients with potential medication-related harm within the ED setting for early pharmacist review.

## Method

A consensus approach was followed using Nominal Group Technique (NGT) methodology to rank the most important criteria for inclusion in a future medication-related harm prioritisation tool for ED use. NGT methodology was selected as it allows all participants to contribute equally and prevents dominance of discussion by individuals [20–22]. This approach is well-suited to defining priority lists and develops structured rankings through facilitated group activity.

### Setting and recruitment of participants

The study was conducted across two ED sites at GCHHS, a metropolitan health service on the Gold Coast, Queensland, Australia: Gold Coast University Hospital (GCUH) and Robina Hospital. GCUH is a tertiary hospital with approximately 900 beds and includes a 108-bed ED, considered one of the busiest in Australia, with an average of 360 patient presentations per day [23]. Robina Hospital has approximately 400 beds and a 49-bed ED. One workshop was held at each site.

Participants were purposively recruited ED clinicians including pharmacists, doctors and nurses who worked at either ED at the time of recruitment with experience in managing patients with potential mediation-related harm. Additional participants were identified through snowball sampling [24], with a target of 5–7 participants per workshop, which is considered the optimal number for NGT [20]. All participants received a participant information and consent form prior to attendance and provided written informed consent.

### Identification of criteria for NGT workshop rankings

A list of criteria was developed through a review of English-language publications in Cochrane, Medline, PubMed, Scopus, Embase and Google Scholar. The review did not include grey literature. Studies were screened by title and abstract, with potentially relevant publications reviewed in full by AF initially and reviewed by LH, KH and GG. Studies including medication-related harm prioritisation tools (ED-based or non-ED), and studies describing factors associated with medication-related harm were included. Searches were first undertaken in June 2024 and repeated in May 2025. Criteria were reviewed and revised by all team members to ensure clarity.

### Data collection

Participants were invited to attend a 1-h workshop, either in person, or some had to join over Microsoft Teams (New Teams 2.x) due to clinical duties. The workshops used a modified NGT process to allow for clinician time constraints and followed a structured format:

1. Introduction and explanation of the criteria list
2. First round ranking—participants selected and ranked their top 12 criteria
3. Group review and discussion of top-ranked criteria
4. Second round ranking—participants ranked their top six criteria from the refined list

Before commencing each workshop, the list of criteria was explained, and the participants had the opportunity to ask questions and clarify criteria. Workshops were facilitated by a senior clinical pharmacist (KH) and involved two rounds. In the first round, the participants ranked their top 12 criteria from the list, with the first being most important and the last being least important. This ranking was done using Slido, an online Q&A polling platform, which participants were able to use on their mobile phones to maintain privacy (Cisco Systems, 2025). Slido assigned a weighted score to each criterion based on its ranking by each individual participant. After the first round, the top 12 criteria based on the group’s combined rankings were displayed to all workshop participants, whilst individual rankings remained anonymous. Participants then had the opportunity to discuss the criteria, suggest and agree on changes. Following this discussion, a second ranking round was held in which participants rated their top six criteria from the group’s top 12 criteria, following the same ranking system as the first round with one as the most important criterion and six the least important. While many NGT exercises report the top three to five priorities, in health and implementation research NGT is frequently used to identify broader sets of priorities to inform staged decision-making and system-level planning [25, 26].

The workshops were recorded on Microsoft Teams to ensure all important information during the workshops was captured. Two research team members (AF & LH) made field notes. The recordings and field notes were reviewed to confirm data captured during workshops were accurate.

### Data analysis

Participant rankings were converted into weighted scores (rank 1 = 12 down to rank 12 = 1, with unranked criteria scored 0) and summed to produce overall rankings. Top criteria were identified within each group and across both workshops. Finally, the scores of highest-ranked criteria across workshops were combined to generate the final consensus list. Inter-rater agreement between individual workshop’s participant was assessed using Kendall’s Coefficient of Concordance (W), appropriate for ordinal rankings from multiple independent raters assessing the same items, within workshop participant groups [27]. A value of 0 indicates no agreement, while a value of 1 indicates perfect agreement. Values above 0.9 are considered to indicate “very good” agreement. Additional adjustments were not performed as all of the raters ranked all criteria.

### Ethics approval

This study was performed in line with the principles of the Declaration of Helsinki. Ethics approval for this study was obtained from the GCHHS and Griffith University Human Research Ethics Committees (Reference (LR) HREC/2025/QGC/116152).

## Results

### Participant details

A total of 14 participants participated, seven each from GCUH and Robina Hospital. The Robina Hospital workshop took place on Wednesday 28 May 2025, and the GCUH workshop on Wednesday 4 June 2025. Robina Hospital workshop participants were an advanced pharmacist (team leader), two senior pharmacists, two registered nurses, a nurse practitioner, and an ED consultant. GCUH workshop participants were three senior pharmacists, a registered nurse, an ED consultant, a clinical facilitator, and a general medicine staff specialist.

### Criteria

A list of 150 criteria was established following the literature review. This list was refined to only include medication specific criteria. Therefore, it only included (1) specific medications or medication classes and (2) general health and medication management criteria. Criteria relating to patient (e.g. ethnicity) or clinician characteristics (e.g. history should be taken by a pharmacist), comorbidities and symptoms (e.g. diabetes, pain), and general admissions information (e.g. after-hours admission) were excluded. Certain medications were condensed under therapeutic classes. Criteria relying on pathology test results were also removed as this information is not available at the start of the admissions process. Following this process a final list of 34 criteria was created. This list was split into the two separate categories described above (see Online Appendix 1). Patient characteristics such as age and gender were not included as this information is routinely already collected as part of the ED presentation.

### Round 1 rankings

**Fig. 1:**
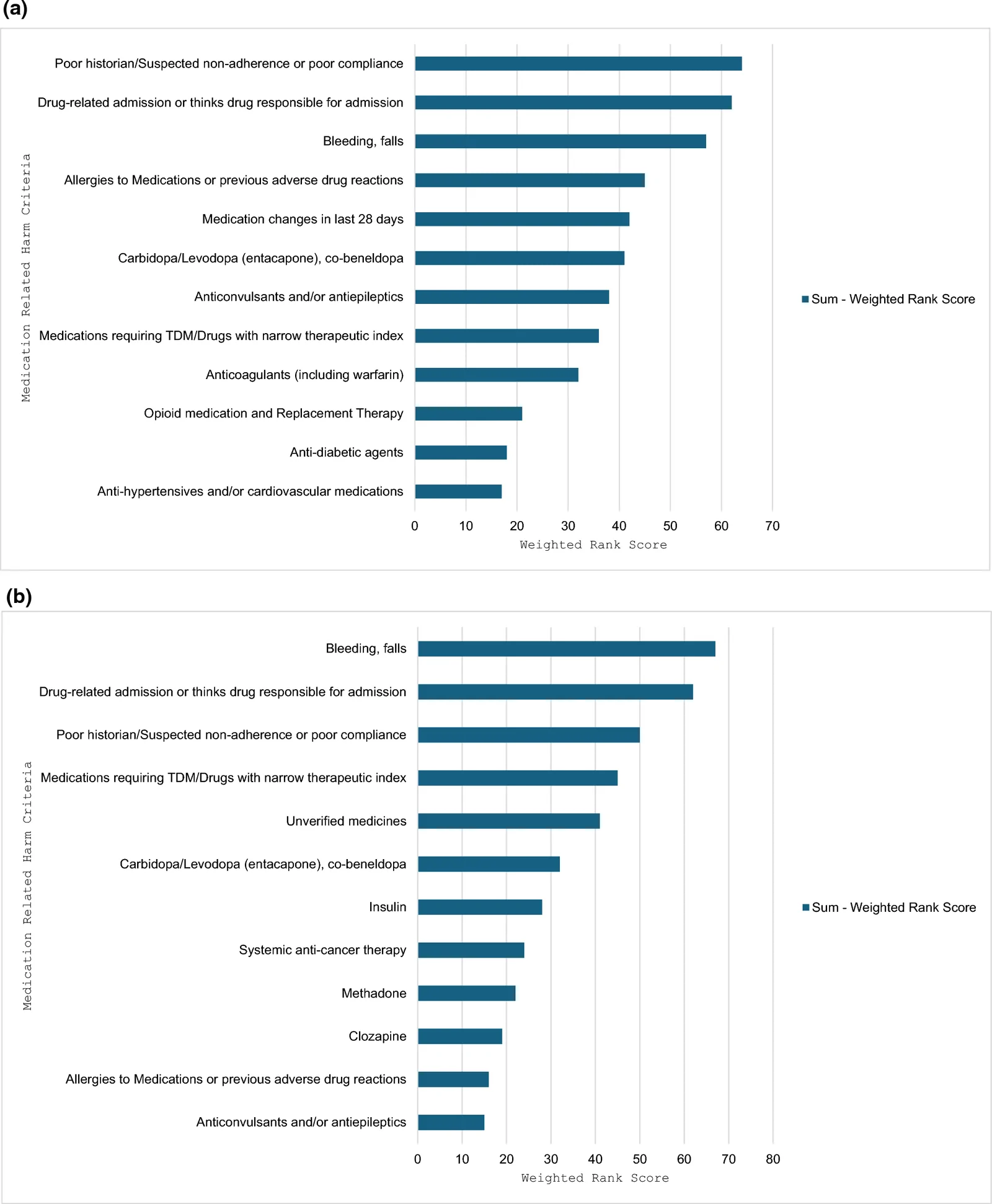
**a** Top 12 criteria from first voting round for Robina Hospital workshop. **b** Top 12 criteria from first voting round for Gold Coast University Hospital workshop

Figures 1a and b show the top 12 criteria from both Round 1 rankings following the initial discussions at the start of the workshops with some agreed changes (e.g. changing of criterion methadone to opioid replacement therapy and combining opioid medications and opioid replacement therapy into one criterion).

Eight criteria were in the top 12 for both workshops: (1) allergies to medications or previous adverse medication reactions, (2) anticonvulsants and/or antiepileptics, (3) bleeding and falls, (4) carbidopa/levodopa (entacapone), co-beneldopa, (5) medication-related admission or thinks medicine responsible for admission, (6) medicines requiring therapeutic drug monitoring/medicines with narrow therapeutic index, (7) opioid medication and replacement therapy, and 8) poor historian (unable to provide reliable history)/suspected non-adherence or poor compliance.

Bleeding and falls: Due to the very high number of patients that present to the ED with either bleeding or falls, both workshop groups made it clear that they selected this criterion with the caveat that it was bleeding and falls in context of certain other factors including elderly patients, particularly with polypharmacy; had recently started a new medication; were taking anticoagulants; or were experiencing both falls/a fall and bleeding together.

### Round 2 rankings

**Fig. 2:**
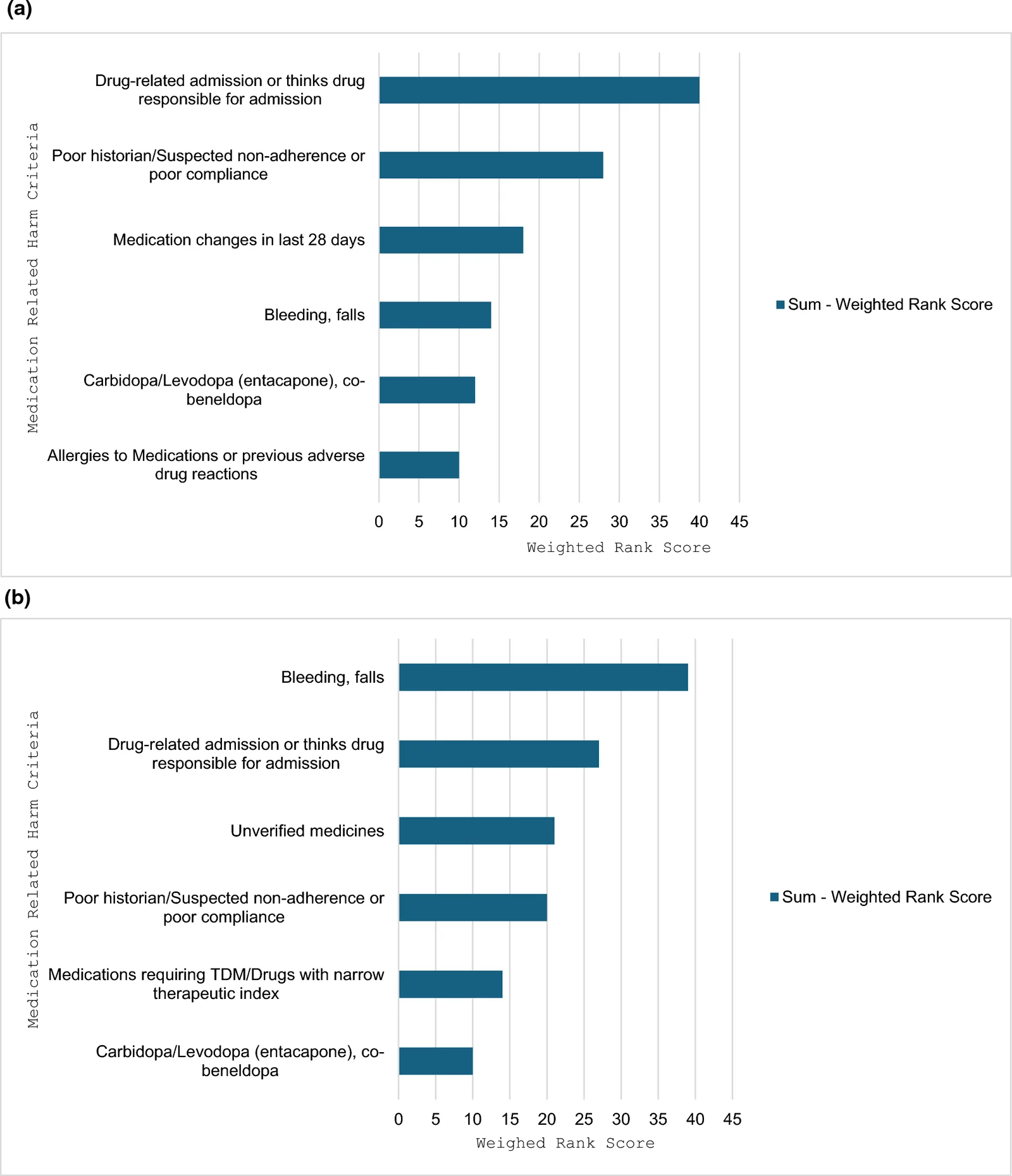
**a** Top six criteria from second voting round at Robina Hospital workshop. **b** Top six criteria from second voting round at Gold Coast University Hospital workshop

Figures 2a and b show the top six criteria from both Round 2 rankings.

### Top rated criteria

**Fig. 3:**
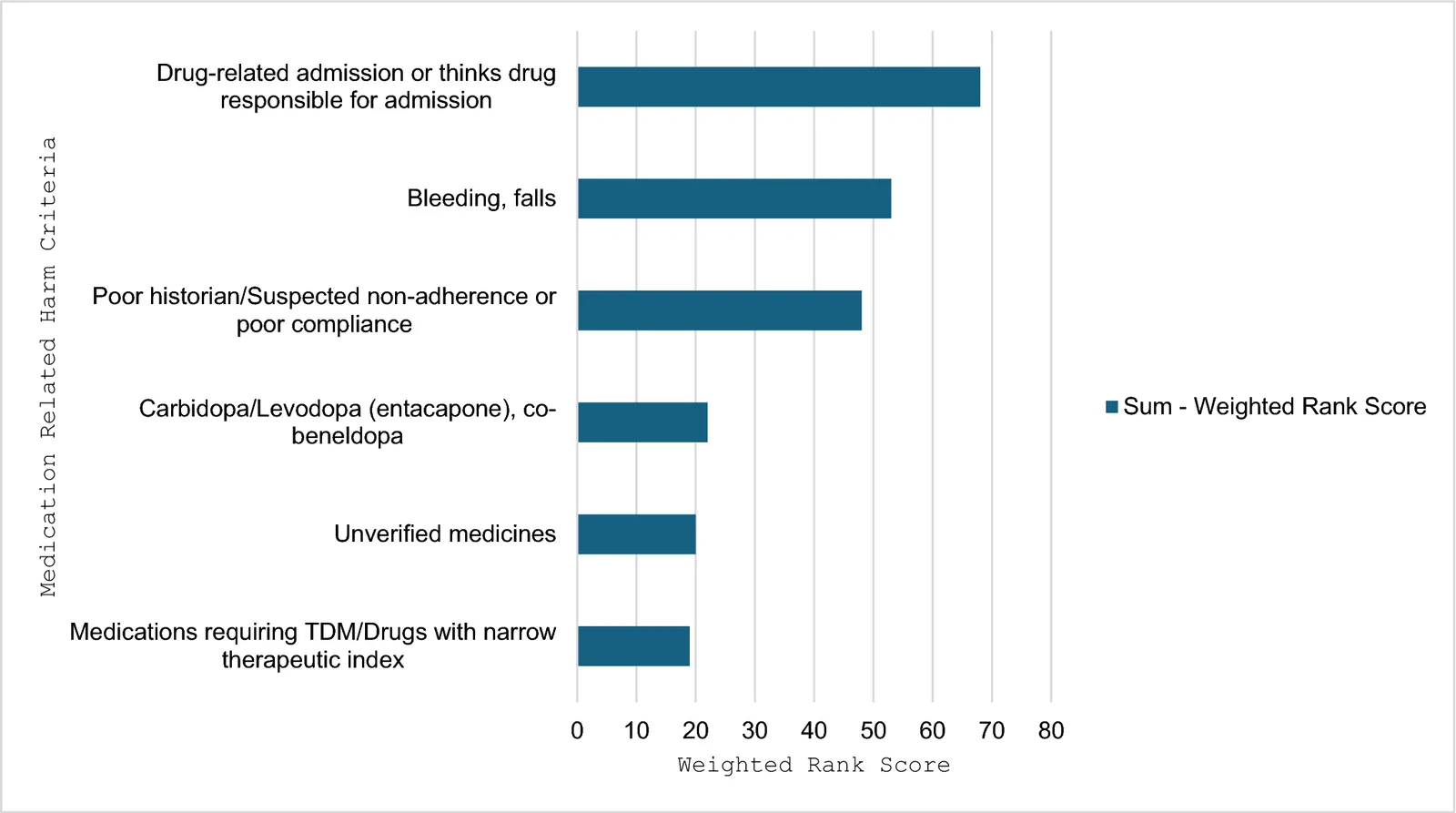
Top six criteria overall

The top six criteria combined from both workshops were (1 = most important) (Fig. 3):

1. Medication-related admission or thinks medicine responsible for admission
2. Bleeding and falls
3. Poor historian *(*unable to provide reliable history*)*/suspected non-adherence or poor compliance
4. Carbidopa/levodopa (entacapone), co-beneldopa
5. Unverified medicines (i.e. unknown or unidentified medicines)
6. Medicines requiring therapeutic drug monitoring (TDM)/medicines with narrow therapeutic index

The first four criteria were ranked within the top six in both workshops. The criteria ‘medicines requiring therapeutic drug monitoring/medicines with narrow therapeutic index’ and ‘unverified medicines’ were only ranked in the top six in the GCUH workshop.

### Level of agreement between participants

The results of the Kendall’s W statistical tests (Table 1) show that there was a moderate level of agreement overall. The agreement between raters was higher in the second round, when selecting from their top 12 criteria, rather than from the full list.

**Table 1:** Level of agreement between participants

Group and Round	Kendall’s W
Robina Hospital, Round 1	0.46
Robina Hospital, Round 2	0.59
Gold Coast University Hospital, Round 1	0.50
Gold Coast University Hospital, Round 2	0.61
Combined, Round 1	0.41

## Discussion

Fourteen ED clinicians developed a list of consensus-based criteria for early pharmacist review of patients presenting to EDs with possible medication-related harm. Participants selected and ranked criteria and the top six criteria, ranked from 1 to 6, were: (1) medication-related admission or thinks medication responsible for admission, (2) bleeding and falls, (3) poor historian (unable to provide reliable history)/suspected non-adherence or poor compliance, (4) carbidopa/levodopa (entacapone), co-beneldopa, (5) unverified medicines, and (6) medicines requiring therapeutic drug monitoring/medicines with narrow therapeutic index. Four criteria overlapped from both workshops, however there were moderate levels of agreement overall.

Patients presenting with a confirmed or suspected admission due to a medicine(s) were rated as the highest priority for pharmacist review. It is not surprising that patients with known or suspected medication-related harm were flagged for priority review. However, ED clinicians are frequently required to make rapid admission decisions in the context of incomplete histories, diagnostic uncertainty, and time pressure, which can make identification of medication-related causes challenging. The likelihood of medication-related admission is increased in patients with recent transitions of care, multiple prescribers, or difficulty providing an accurate medication history. Recognition of these temporal relationships between symptom onset and medication initiation, cessation, or dose adjustment is critical, as is awareness that difficulty obtaining a reliable history may itself indicate increased medication risk. Specific medicines also play a role: data from the Australian National Hospital Morbidity Database on hospital admissions due to all drugs, including alcohol, tobacco, prescription medicines, over-the-counter medicines, and illicit drugs, found 12.9% of medication-related hospital admissions were related to analgesics (including heroin and opium), 7.6% were attributable to sedatives and hypnotics (not including alcohol), and 6.8% were due to antidepressants and antipsychotics in 2016–2017 [28]. ED clinicians need to be familiar with all of these factors that could be linked to medication-related admissions.

Patients who present with bleeding and falls was the second ranked criteria overall and the top ranked criteria by the GCUH workshop participants. Falls Risk Increasing Drugs (FRIDs) can be a significant contributing factor to falls however are considered a modifiable risk factor, and clinician intervention is important in their management, reduction, and deprescribing, which can reduce falls risk [29]. Identifying medications that could contribute to falls early in a patient’s hospital journey is important for the ongoing management of the patient, including whether medication changes may be required. It is clear these patients will benefit from early pharmacist review however workshop participants indicated the falls should be prioritised when associated with bleeding whilst also considering age, polypharmacy, anticoagulant usage, and recent commencement of a new medicine.

Suspected non-adherence or poor medication compliance was ranked third overall. Poor medication adherence and compliance can result in medical conditions worsening, increased comorbidities, and increased mortality, placing a burden on the healthcare system [30]. Pharmacists play an important role in identifying medication-related harm as a result of non-adherence/non-compliance and educate patients to improve adherence [31, 32]. Although this criterion could be challenging when included in a prioritisation tool due to requiring the use of open questions [33], strategies to manage this included better patient engagement through self-recorded medication histories and digital approaches [34, 35].

Specific medication classes were ranked fourth and sixth: carbidopa/levodopa (entacapone)/co-beneldopa and medicines requiring therapeutic drug monitoring/medicines with narrow therapeutic index. Carbidopa/levodopa/*co-*beneldopa are used in the treatment of Parkinson’s Disease (PD). Patients with PD face disproportionate rates of preventable and unintended harm in the acute care hospital setting with specific challenges when medication doses are omitted that can lead to deterioration of symptoms within a short time period [36]. Patients with PD often require complex dose regimens including specific dose timing and there is a multitude of available brands which can leading to potential prescribing and/or administration errors that specifically cause risk to these patients during transition of care. Medications with a narrow therapeutic index that require monitoring have been shown to contribute to ED presentations in various studies [37, 38]. Identifying these patients for priority pharmacist review will facilitate identification of non-optimal dosing and avoid potential drug-drug interactions.

We identified tools for the prioritisation of ED patients with potential medication-related harm however there was little overlap between the criteria included in the various tools [11–18, 39]. These tools included both medication related criteria and sociodemographic factors such as concession status with some including particular disease states. We aimed to develop a list of consensus-based criteria to inform information to be gathered by clinicians. Once the criteria have been further refined it will be incorporated into a digital prioritisation tool. Considering that some criteria may not be available from patients’ electronic medical records early in their ED admission, further work is required to explore whether obtaining the information could be incorporated into workflow processes, and in co-designing new workflow procedures, roles and responsibilities to obtain missing medication information. The workshop rankings showed substantial overlap in the top ranked criteria between both workshop groups, hence there was consensus across the two groups. However, there was variation in the specific rankings given by individual participants, which accounts for moderate level of agreement shown by the Kendall’s W statistical tests, and which also warrants further refinement of criteria.

A strength of our study is that the participants were all experienced ED clinicians from two hospitals and covered medical, pharmacy and nursing professions. Two workshops were conducted which allowed for a comparison between the two workshop groups. A limitation of our study was time constraints on clinicians which impacted recruitment, and necessitated limiting the workshops to one hour. Hence a decision was made to focus on medication-related criteria. We acknowledge that participants’ understanding of the timeframe to collect the additional information could have impacted their ratings. To address this, the explanation at the start of the workshops clarified that participants had to consider criteria required early (within the first six hours) during patients’ ED admission. There were moderate levels of agreement between participants from the same workshop and two of the criteria were from one hospital workshop only. This limits the generalisability of the results as more research is required to develop criteria with stronger levels of agreement. This study was conducted in one Australian metropolitan hospital and health service and findings may not be transferable to hospitals within other settings. The results may also not be transferable to other countries, especially those with different ED staff roles and responsibilities and settings that do not have digital medical record systems in place.

## Conclusion

This study generated a concise set of six medication-related criteria to identify patients at risk of medication-related harm. The agreed criteria provide a platform for the next phase of work, enabling meaningful engagement with clinicians, operational leaders, and key stakeholders. These discussions will guide the refinement and practical integration of the criteria into workflows and a medication-related harm prioritisation tool tailored specifically for use in the ED. Ultimately, this work aims to support more timely intervention, enhance patient safety, and streamline clinical decision-making in this high-pressure setting.

## Supplementary Information

Below is the link to the electronic supplementary material.

Supplementary file1 (DOCX 18 KB)

## Data Availability

No datasets were generated or analysed during the current study.
