# Supplementary material for: Prioritising medication management criteria to identify emergency department patients with potential medication-related harm: Nominal Group Technique workshops with clinicians
Source: Int J Clin Pharm. 2026 Jul 31;48(5):2139–48. doi: 10.1007/s11096-026-02199-9 (PMC13569595; doi:10.1007/s11096-026-02199-9)
Supplement: Supplementary file 1 [file 11096_2026_2199_MOESM1_ESM.docx]

Supplementary File: List of criteria from the literature

| **General Health and Medication Management** | | |
| --- | --- | --- |
| 1. Allergies to medications or ADRs | 6. Person administering medication has difficulty reading labels | 11. Taken antibiotics in last 7 days |
| 2. Bleeding and falls | 7. Uses dose aid | 12. Medication changes in last 28 days |
| 3. Medication-related admission or thinks medication responsible for admission | 8. Drug misuser | 13. Medications requiring therapeutic drug monitoring/medicines with narrow therapeutic index |
| 4. Poor historian/Suspected nonadherence or poor compliance | 9. Number of prescription medications (specify number) | 14. Unverified medicines |
| 5. Self-administered or administered by carer | 10. Has taken medication in the last 24 hours | 15. Taking a high-cost medicine |
| **Specific Medications** | | |
| 16. Anti-hypertensives and/or cardiovascular medications | 23. Phenytoin | 30. Insulin |
| 17. Digoxin | 24. Carbidopa/levodopa (entacapone), co-beneldopa | 31. NSAIDs |
| 18. Antipsychotics | 25. Anticoagulants (including Warfarin) | 32. Hormonal preparations |
| 19. Lithium | 26. Systemic antimicrobials | 33. Systemic anti-cancer therapy |
| 20. Clozapine | 27. Opioid medications | 34. Zolpidem |
| 21. Haloperidol | 35. Methadon |  |
| 22. Anticonvulsants and/or antiepileptics | 36. Anti-diabetic agents |  |
